# The important role of comorbidities in the management of obesity

**DOI:** 10.1210/jendso/bvag012

**Published:** 2026-01-20

**Authors:** Sarah R Barenbaum, Beverly G Tchang, Louis J Aronne

**Affiliations:** NewYork Presbyterian Hospital/Weill Cornell Medical College, Comprehensive Weight Control Center, Division of Endocrinology, Diabetes & Metabolism, New York, NY 10021, USA; NewYork Presbyterian Hospital/Weill Cornell Medical College, Comprehensive Weight Control Center, Division of Endocrinology, Diabetes & Metabolism, New York, NY 10021, USA; NewYork Presbyterian Hospital/Weill Cornell Medical College, Comprehensive Weight Control Center, Division of Endocrinology, Diabetes & Metabolism, New York, NY 10021, USA

**Keywords:** obesity, obesity comorbidities, obesity complications, complications of obesity, weight loss

## Abstract

Obesity is a chronic, multifactorial disease associated with more than 200 complications including type 2 diabetes, cardiovascular disease, obstructive sleep apnea, dyslipidemia, hypertension, premature mortality, and numerous other adverse effects spanning multiple organ systems. In addition to these health complications, obesity also contributes to weight stigma and bias, which negatively affect access to care, treatment outcomes, and quality of life. Together, these complications and social consequences drive high morbidity, premature mortality, and increasing healthcare costs. Early diagnosis of obesity and systematic screening for related conditions are essential to improving health outcomes. Clinicians must treat obesity in addition to managing its complications, offering individualized strategies that may include lifestyle modification, pharmacotherapy, and bariatric surgery. Because obesity is a chronic and relapsing disease, effective management requires long-term follow-up and coordinated care through a multidisciplinary team. The effective treatment of obesity and its comorbidities can improve individual health outcomes, reduce healthcare costs, and reduce the global burden of chronic disease.

Obesity is a complex, chronic, multifactorial disease associated with over 200 complications [1]. Obesity contributes to major health conditions through metabolic or musculoskeletal consequences including type 2 diabetes, cardiovascular disease, obstructive sleep apnea, dyslipidemia, hypertension, premature mortality, and numerous other adverse effects spanning multiple organ systems (Fig. 1) [2]. Obesity is also associated with stigma and bias, which reduces quality of life and increases morbidity [3]. The prevalence of obesity has increased significantly since 1990, which has led to an increased number of patients experiencing complications associated with excess weight [4]. The World Health Organization has estimated that over 1 billion people globally have obesity [4]. More than 40% of American adults have obesity, and by 2030 nearly 50% of American adults are projected to have obesity [5, 6].

**Figure 1:**
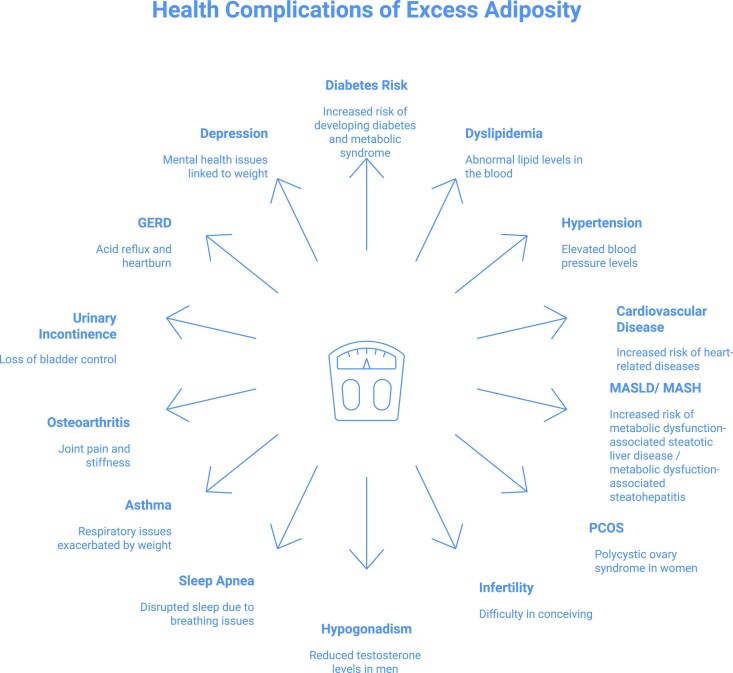
Health complications of excess adiposity. This figure illustrates some of the common complications of excess adiposity.

The relationship between excess adiposity and metabolic disease has been known for decades with initial nomenclature sufficing as “metabolic syndrome” or “syndrome X” [7]. In 2008 the Obesity Society published a white paper arguing for obesity as a disease, and in 2012 the American Association of Clinical Endocrinologists issued a similar position statement. Finally in 2013, the American Medical Association officially recognized obesity as a disease [8]. However, perhaps due to stigma, bias, limited time allotted with patients and limited provider acceptance of obesity as a disease, clinicians have traditionally focused on managing the complications of excess weight rather than addressing obesity itself [9]. A greater acceptance that obesity is a disease and not a lifestyle choice is beginning to take hold as providers better understand that obesity is the root cause of downstream comorbidities [9, 10]. This recognition has shifted clinical practice toward treating obesity directly in addition to its complications, since treating obesity leads to improvement or resolution of the comorbidities. While a full discussion of all the obesity-related conditions is outside the scope of this paper, this mini-review highlights the importance of obesity-related comorbidities, which collectively drive morbidity, mortality, and healthcare burden. This paper also highlights effective management strategies that are essential because the comprehensive treatment of obesity is the treatment of all metabolic disease.

## Materials and methods

Several electronic databases were searched for this review including PubMed, Web of Science, Scopus, and Cochrane Library for relevant literature published through August 2025. The literature search was conducted using combinations of medical subject heading terms such as obesity, obesity comorbidities, obesity complications, etc. The search was limited only to articles in English.

## Diagnosis of obesity

Body mass index (BMI), which is the weight in kilograms divided by the height in meters squared, is used to diagnose and classify obesity on the population level with a BMI ≥30 kg/m^2^ considered obesity. Classes of obesity (class I: BMI 30-34.9 kg/m^2^, class II: BMI 35-39.9 kg/m^2^, and class III: BMI ≥40 kg/m^2^) are associated with increasing levels of morbidity and mortality. An increasing number of professional societies are recommending lower BMI thresholds for the diagnosis of overweight and obesity in certain populations, for example Asians, who are at an increased risk of cardiometabolic disease at a lower BMI [11, 12].

However, BMI is an incomplete measurement of overall health risk for the individual and should not be used as the sole indicator to diagnose overweight or obesity in the clinical setting. BMI is a measure of mass and does not differentiate between lean and fat mass or directly measure adiposity [13]. It also does not distinguish body fat distribution or function and can over or underestimate cardiometabolic risk in particular populations [14]. BMI also does not consider overall health or the presence of weight related complications. Therefore, in the clinical setting additional anthropometric measures (eg, waist circumference, waist-to height ratio, or waist-to hip ratio) and the presence of any associated health consequences including physical, metabolic, or psychological, are recommended to better evaluate obesity and associated risks [15, 16]. Waist-to-height ratio (WHR), in particular, is gaining popularity because of its superiority to BMI for predicting cardiovascular risk in individuals with BMI 25-29.9 who may already have complications of obesity despite overweight BMI [17]. The WHR is the waist circumference, measured at the midpoint between the bottom of the last rib and the top of the iliac crest, divided by a person's height. WHR of <0.5 is normal, while 0.5-0.59 indicates elevated health risk, and ≥0.6 indicates high health risk. WHR should be measured in individuals with BMI <35 because those with BMI ≥35 are already presumed to have an elevated WHR. Further, in recognizing excess fat mass alone may not constitute a disease, professional organizations have emphasized the importance of obesity-related complication or comorbidity as evidence of harm, thereby establishing the diagnosis of obesity or “clinical obesity” with an anthropometric component and a clinical component [18, 19].

## Pathophysiology linking obesity to comorbidities

The pathophysiology linking obesity to its associated comorbidities is complex and multifactorial and involves metabolic, inflammatory, mechanical, and neuroendocrine mechanisms. Excess adipose tissue is an active endocrine organ that secretes adipokines and proinflammatory cytokines which drive low grade systemic inflammation. This pro-inflammatory and prothrombotic environment contributes to insulin resistance, endothelial dysfunction, and dysregulation of lipid metabolism, which are central to the pathogenesis of type 2 diabetes, atherosclerosis, and metabolic dysfunction-associated steatotic liver disease (MASLD) [20]. Adipose tissue inflammation, marked by macrophage infiltration and a predominance of pro-inflammatory M1 macrophages, leads to additional metabolic dysfunction and further increases the risk of cardiovascular, hepatic, and musculoskeletal complications [20, 21]. Genetic factors are also important contributors to individual susceptibility to obesity and obesity comorbidities, with estimates ranging from 40-70% with respect to the heritability of obesity [22]. Polygenic causes and rare monogenic causes and syndromes can influence appetite regulation, energy balance, insulin resistance, and comorbidity risk. While a full discussion of genetics is beyond the scope of this review, recognizing genetic predisposition and rare monogenic obesity is important in understanding phenotypic variability and comorbidity risk.

With weight gain, ectopic adipose deposition can occur primarily in the liver, kidney, pancreas, and heart which directly contributes to organ dysfunction. For example, ectopic adipose deposition can lead to renal hyperfiltration with progression to chronic kidney disease or end stage kidney failure, development of arrythmias or nonischemic heart failure, and MASLD [1]. Within adipocytes, excess lipid storage and release of free fatty acids into circulation also contribute to the development of dyslipidemia, coronary artery disease, lipotoxicity, and MASLD with progression to metabolic dysfunction association steatohepatitis and cirrhosis. Excess weight also leads to the development of hyperleptinemia and leptin resistance, which can promote cardiovascular dysfunction [23]. Leptin also stimulates aldosterone secretion, which leads to increased activation of the sympathetic nervous system and of the renin-angiotensin-aldosterone system, both of which promote systemic and pulmonary hypertension [24, 25]. Elevated aldosterone activity is frequently observed in obesity and metabolic syndrome and represents an additional contributor to both hypertension and insulin resistance in individuals with obesity [25].

Notably, sex-specific differences in adipose tissue distribution can significantly influence cardiometabolic risk. Women typically accumulate more subcutaneous fat while men accumulate more visceral adiposity, which confers greater metabolic risk [26]. Among women with polycystic ovary syndrome, those with insulin-resistant and obesity-associated phenotypes—particularly when hyperandrogenism is present—exhibit worse cardiometabolic profiles and increased cardiovascular risk relative to women with nonhyperandrogenic polycystic ovary syndrome [27]. Pregnancy also represents a unique cardiometabolic stressor where obesity substantially increases risks for gestational diabetes, hypertensive disorders, and adverse perinatal outcomes [28]. In the evaluation of weight-related comorbidities, therefore, it is important to consider sex-specific differences that can confer additional patient risk.

Excess weight also leads to significant mechanical complications including osteoarthritis by increasing joint load and obstructive sleep apnea/obesity hypoventilation syndrome by increasing pharyngeal soft tissue and alteration of the anatomy of the upper airway. Increased intrabdominal pressure can result in gastroesophageal reflux disease, Barrett's esophagus, and esophageal adenocarcinoma [24].

Obesity is also associated with increased prevalence of psychiatric conditions including depression, anxiety, and other mood disorders. The pathophysiology is complex and likely bidirectional as obesity can predispose to depression though biological and psychosocial pathways, and depression can worsen obesity through neuroendocrine and behavioral effects and the weight-promoting effects of many antidepressant medications [29, 30].

## Clinical impact of comorbidities

The clinical impact of obesity and its associated complications is profound and multifaceted. Obesity is one of the most important modifiable risk factors in the development of type 2 diabetes. According to data from the National Health and Nutrition Examination Survey from 1999 to 2006, the prevalence of diabetes increased with increasing BMI, ranging from 8% in individuals of normal weight to 43% among those with class III obesity [31]. Similarly, obesity is associated with higher rates of cardiovascular events. In a pooled cohort of adults aged 40 to 59 years with a total follow-up of 856 523 person-years, cardiovascular event rates were higher among individuals with obesity. Men with a BMI between 30 and 39.9 experienced 20.21 events per 1000 person-years compared with 13.72 events among men with a normal BMI. Similarly, women in the same BMI range experienced event rates of 9.97 per 1000 person-years vs 6.63 in women with a normal BMI [32]. Taken together, these examples illustrate that obesity is a driver of multiple chronic diseases, with risk escalating in a dose-response manner as BMI increases. Among individuals with obesity, greater severity of excess weight is associated with higher levels of complex multimorbidity, showing a clear dose–response pattern [33]. In a cross-sectional study of 270 657 participants in the All of Us research program, obesity was correlated with 16 diseases: hypertension, type 2 diabetes mellitus, hyperlipidemia/dyslipidemia, heart failure, atrial fibrillation, atherosclerotic cardiovascular disease, chronic kidney disease, pulmonary embolism, deep vein thrombosis, gout, MASLD, biliary calculus, obstructive sleep apnea, asthma, gastroesophageal reflux disease, and osteoarthritis [34]. Class III obesity conferred the strongest associations, with an 11-fold increased risk of obstructive sleep apena and a 7-fold increased risk of diabetes and MASLD. Epidemiologic studies examining patient-centered outcomes such as pain and fatigue have revealed similar relationships. Among 323 640 participants in the All of Us program, higher BMI was associated with severe pain and fibromyalgia, with class III obesity again demonstrating the most pronounced effect with a 4-fold increased risk of severe pain and a 3-fold increased risk of fibromyalgia [35]. All of these comorbidities contribute to increased morbidity, premature mortality, and reduced quality of life.

Another major clinical impact of obesity that is perhaps more insidious but equally harmful is the weight stigma and bias that individuals with obesity encounter. Up to 40% of American adults reported experiencing some form of weight stigma and bias [36]. In studies, physicians have indicated they would spend less time with patients who have obesity than those with normal weight. Furthermore, 10.3% of American adults report having experienced weight bias in healthcare [37]. Weight stigma contributes to delayed diagnosis, reduced access to care, and worsened clinical outcomes. It also leads to negative psychosocial, psychological, and employment outcomes, ultimately decreasing quality of life and overall well-being [38].

Obesity and its related conditions also lead to higher healthcare utilization and costs. The costs associated with obesity include both direct and indirect costs. Direct costs include the medical expenses of obesity and its related conditions, but these only represent a fraction of the overall economic burden of excess weight. Direct costs include expenses for medical visits, hospitalizations, diagnostic testing, medications, and procedures, along with the ongoing costs of managing obesity-related complications and coordinating care through a multidisciplinary team. The indirect costs are harder to identify and quantify but include lost economic opportunity and productivity due to disability, absenteeism, presenteeism, lost wages and lower probability of employment, and premature mortality [39]. The Milken institute reported that in 2016 the chronic diseases associated with obesity lead to $480.7 billion in direct healthcare costs in the United States and roughly $1.24 trillion in indirect healthcare costs. Together, these costs approached $1.72 trillion, representing close to 9.3% of the nation's gross domestic product in 2016 [40].

## Screening and diagnosis of the complications associated with excess adiposity

Early recognition and comprehensive management of obesity and its related conditions can greatly improve outcomes of obesity and can reduce the downstream impact of the complications. This can be done with systematic screening for comorbidities at diagnosis and during follow-up, which is important to guide both treatment and referrals. Annual screening and symptom-based evaluations should be conducted for the major complications associated with obesity including type 2 diabetes, hypertension, hyperlipidemia, cardiovascular disease, obstructive sleep apnea, osteoarthritis, MASLD, and depression [41]. Physicians must additionally use their clinical judgment when conducting a thorough history and physical exam and when ordering diagnostic testing to evaluate for other possible complications and subsequent management.

## Management implications

The management of obesity must address both the disease itself and its related comorbidities, using lifestyle, pharmacotherapy, and surgical interventions tailored to the individual. The management of obesity and its related conditions requires a patient-centered approach with shared decision-making. Healthcare providers should recognize and address their own implicit and explicit weight biases in order to minimize their impact on patient discussions or treatment [15]. Once a patient has been diagnosed with obesity and screened for associated conditions, the provider should assess the patient's readiness to address their weight before initiating treatment [15]. If the patient is ready to engage in a weight management plan, treatment should be individualized and may require a multidisciplinary team. For those with concern for more advanced complications, a referral to a specialist should be made (eg, if there is concern for metabolic-associated steatohepatitis, a referral to hepatology should be made).

All guidelines for the treatment of overweight and obesity recommend initiating a calorie-reduced diet, exercise, and behavioral changes [12, 41]. For those who qualify, obesity pharmacotherapy and/or bariatric surgery should also be recommended. Bariatric surgery is a powerful tool for lifelong significant weight loss, which can lead to resolution of significant comorbidities and an improvement in mortality (Table 1) [71]. However, there are no large randomized controlled trials specifically comparing long-term outcomes including weight loss, comorbidity resolution, or mortality of bariatric surgery vs nutrient-stimulated hormone medications (eg, incretin hormone mimetics). Therefore, patients should be offered all options and the decision of whether to pursue bariatric surgery vs pharmacotherapy, or a combination of the 2 should be individualized to their preferences and goals.

**Table 1:** Obesity medications and bariatric surgery with secondary benefits

	Orlistat	Phentermine/topiramate	Naltrexone/bupropion	Liraglutide 3.0 mg	Semaglutide 2.4 mg	Tirzepatide	Bariatric surgery
T2DM, prediabetes [42]	✓	✓	✓	✓	✓	✓	✓ [43]
Dyslipidemia, hypertriglycer-idemia [42]	✓	✓	✓	✓	✓	✓	✓ [43]
Hypertension [42]	✓	✓	✓	✓	✓	✓	✓ [43]
CVD					✓ [44]	✓ [45]*^a^*	✓ [46, 47]
MASH				✓ [48]	✓ [49]		✓ [50]
PCOS	✓ [51]			✓ [52]	✓ [53]		✓ [54]
Female infertility					✓ [53]		✓ [55]
Male hypogonadism					✓ [56]	✓ [56]	
OSA		✓		✓ [57]		✓ [58]	✓ [59]
Osteoarthritis				✓ [60]	✓ [61]		✓ [62]
Urinary stress incontinence							✓ [43]
GERD							✓*^b^* [63]
Depression or anxiety			✓ [64]	✓ [65]	✓ [66]	✓ [67]	
Cancer				✓ [68]	✓ [68]	✓ [68]	✓ [43]
CKD					✓ [69]		✓ [70]

Abbreviations: CKD, chronic kidney disease; CVD, cardiovascular disease; GERD, gastroesophageal reflux disease; MASH, metabolic dysfunction-associated steatohepatitis; OSA, obstructive sleep apnea; PCOS, polycystic ovary syndrome; T2DM, type 2 diabetes mellitus.

^*a*^This is based on the SURPASS-CVOT trial, a cardiovascular outcome trial for patients with T2DM and established atherosclerotic cardiovascular disease who were treated with tirzepatide. The cardiovascular outcome trial for patients with obesity, SURMOUNT MMO, is ongoing.

^*b*^Specifically the Roux-en-y gastric bypass.

The therapeutic choice of which pharmacologic agent to start should be individualized and guided by each patient's preferences, medical history, contraindications to specific agents, drug-drug interactions, and insurance coverage since unfortunately access to medication options vary based on insurance plans [72]. Furthermore, some obesity medications have additional Food and Drug Adminstration (FDA) indications to treat related conditions (eg, tirzepatide is approved to treat obesity, type 2 diabetes, and obstructive sleep apnea). Notably, all obesity medications demonstrate actual or potential benefit in treating other cardiometabolic or musculoskeletal complications (Table 1).

For patients with obesity and related comorbidities, pharmacotherapy should, when possible, be selected to address both the excess weight and the associated condition. For example, in a patient who has obesity, type 2 diabetes, and obstructive sleep apnea, tirzepatide would be an appropriate choice since it is FDA approved to treat all 3 conditions. If a patient has obesity and MASLD, semaglutide would be an appropriate choice since it is FDA approved to treat both conditions.

**Figure 2:**
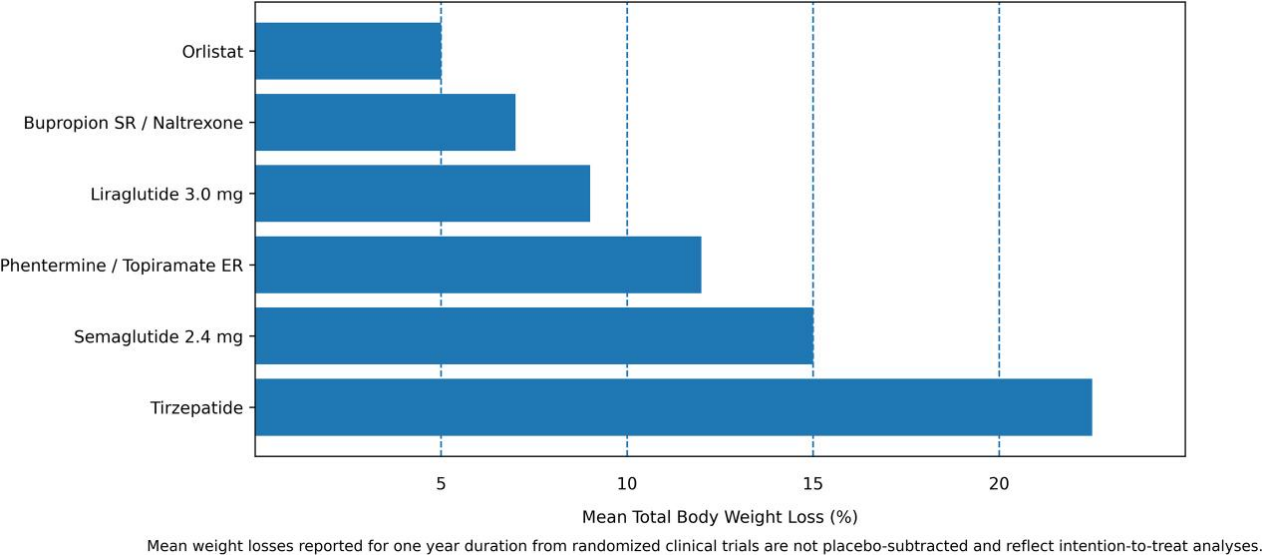
Mean weight loss achieved with long-term obesity medications (not placebo-subtracted. This figure illustrates the mean total body weight loss reported in clinical trials for Food and Drug Administration-approved obesity pharmacotherapy.

The goal of weight loss is individualized but should be focused on health and remission of the associated conditions rather than on achieving a “normal” BMI. There is a dose-response relationship between the amount of weight loss achieved by lifestyle interventions and the improvement in metabolic parameters. For example, with as little as 3% to 5% total body weight loss, there are reductions in fasting glucose, hemoglobin A1c, and triglycerides [73]. With 5% to 10% weight loss, there is improvement in dyslipidemia, hypertension, osteoarthritis and stress urinary incontinence [12, 73]. With 10% to 15% weight loss, there is improvement in cardiovascular disease, obstructive sleep apnea, and metabolic dysfunction-associated steatohepatitis [12]. With 15% weight loss and more, there can be remission of type 2 diabetes, remission of metabolic dysfunction-associated steatohepatitis, and an improvement of cardiovascular mortality [73]. Greater weight loss produces greater benefits. Considering the amount of weight loss needed to see an improvement in a patient's comorbidities is another way to decide which obesity pharmacotherapy to initiate. For example, all obesity medications lead to 5% weight loss, which can be targeted for prevention of incident diabetes, while highly effective pharmacotherapies such as semaglutide and tirzepatide can access greater degrees of weight loss (15-20%), which is associated with significant disease improvement or remission (Fig. 2) [74]. Therefore, the overall goals of weight loss should be individualized through shared decision-making based on the patient's goals considering comorbidities as well.

Importantly, patients require lifelong follow-up and management of obesity and its related conditions, especially following significant weight loss. Maintaining weight loss is particularly challenging due to adaptive physiologic processes that favor weight regain [75, 76, 77]. Obesity medications are therefore considered lifelong, and randomized withdrawal studies of obesity medications consistently demonstrate weight regain when pharmacotherapy is discontinued [78, 79]. Despite guidelines and evidence recommending long-term use of obesity pharmacotherapy, there is poor provider awareness or uptake of these recommendations, little consensus on the total duration of therapy required to treat obesity, and limited long-term patient adherence to obesity medications [12, 80, 81]. Weight regain is clinically important because it can reverse the health benefits achieved through weight loss [82]. Obesity must be treated like any other chronic disease with long-term follow-up and management.

## Conclusion

Obesity is a complex, chronic disease that drives a wide spectrum of comorbidities spanning all body systems. These complications lead to substantial morbidity, impaired quality of life, premature mortality, and escalating healthcare costs. The recognition of obesity as the underlying cause of these conditions is essential, and it is imperative that clinicians shift from managing downstream consequences alone to treating obesity directly. Early identification, systematic screening, and comprehensive management are essential to reduce the burden of disease. Addressing stigma, bias, and barriers to care is also critical to ensure equitable access to effective treatment. Ultimately, treating obesity as a chronic disease with long-term, holistic multidisciplinary care with a patient-centered framework has the potential to improve individual health outcomes, decrease healthcare expenditures, and lessen the burden of chronic disease.

## Data Availability

Data sharing is not applicable to this article as no datasets were generated or analyzed during the current study.
